# Von der Extrasystole zur anhaltenden Kammertachykardie: Neuerungen der ESC-Leitlinie 2022 zur Therapie ventrikulärer Arrhythmien im Überblick

**DOI:** 10.1007/s00399-022-00908-1

**Published:** 2022-11-16

**Authors:** Hilke Könemann, Lars Eckardt

**Affiliations:** Klinik für Kardiologie – Rhythmologie, Universitätsklinikum Münster, Albert-Schweitzer-Campus 1, 48149 Münster, Deutschland

**Keywords:** ESC, Leitlinie, Kammerflimmern, Plötzlicher Herztod, Ventrikuläre Tachykardie, ESC, Guideline, Ventricular fibrillation, Sudden cardiac death, Ventricular arrhythmias

## Abstract

Die aktuelle Leitlinie der europäischen Gesellschaft für Kardiologie 2022 zum Management von Patienten mit ventrikulären Arrhythmien und zur Prävention des plötzlichen Herztods aktualisiert die Leitlinie aus dem Jahr 2015. Mit zahlreichen Übersichtstabellen, Algorithmen und einer umfangreichen Einbeziehung der zugrundeliegenden Studiendaten liegt ein anwenderbezogenes Nachschlagewerk für die klinische Praxis vor, das auch besondere klinische Situationen wie Herzrhythmusstörungen in der Schwangerschaft oder im Zusammenhang mit Sport umfasst. In der Akuttherapie ventrikulärer Arrhythmien ist die Kardioversion auch bei hämodynamisch tolerierter Arrhythmie aufgewertet, zudem liegt ein besonderer Schwerpunkt der Leitlinie auf dem Management des elektrischen Sturms. In der Langzeittherapie sind die Empfehlungen zur medikamentösen Therapie an aktuelle Herzinsuffizienzleitlinien angeglichen. Katheterinterventionelle Verfahren gewinnen nicht nur bei rezidivierenden ventrikulären Tachykardien unter Amiodarontherapie und als Alternative zur ICD-Implantation bei ausgewählten Patienten mit koronarer Herzerkrankung, sondern insbesondere bei der Behandlung idiopathischer ventrikulärer Extrasystolen und Tachykardien an Bedeutung. Die Risikostratifikation bzw. Kriterien zur primärprophylaktischen ICD-Implantation sind unverändert kontroverse Themen, die in der aktuellen Leitlinie anhand der spezifischen Krankheitsbilder ausführlich diskutiert werden.

## Abstract (en)

The recent 2022 European Society of Cardiology Guidelines for the management of patients with ventricular arrhythmias and the prevention of sudden cardiac death are an update of the former 2015 European guidelines. With multiple tables, algorithms, and comprehensive integration of underlying study data, the new guideline is a user-oriented reference book for clinical practice that also covers special clinical situations such as cardiac arrhythmias in pregnancy or in the context of sports. Regarding the acute treatment of ventricular arrhythmias, cardioversion is now recommended in case of hemodynamically tolerated arrhythmias. Beyond that, the guideline places special emphasis on the management of the electrical storm. In long-term therapy, recommendations for drug therapy have been aligned with current heart failure guidelines. Catheter ablation of ventricular arrhythmias has gained importance not only for recurrent ventricular tachycardia under chronic amiodarone therapy and as an alternative to implantable cardioverter–defibrillators (ICDs) in selected patients with coronary artery disease, but especially for the treatment of idiopathic premature ventricular contractions and tachycardias. Risk stratification and criteria for primary preventive ICDs are still controversial topics, which are discussed in detail based on the specific disease entities.

Ventrikuläre Arrhythmien stellen eine häufige Ursache für Morbidität und Mortalität bei Herzerkrankungen dar. In Europa beträgt die jährliche Inzidenz des plötzlichen Herztods etwa 40 pro 100.000 Einwohner [1], wovon etwa die Hälfte der Fälle auf ventrikuläre Tachykardien oder Kammerflimmern zurückzuführen ist [2]. Der plötzliche Herztod ist oftmals die Erstmanifestation einer bis dahin unbekannten Herzerkrankung. Ausgehend hiervon lassen sich zwei Schwerpunkte im Management ventrikulärer Arrhythmien formulieren: einerseits die Akut- und Langzeittherapie symptomatischer Arrhythmien und andererseits die prophylaktische Therapie zum Schutz vor anhaltenden Tachykardien und/oder einem plötzlichen Herztod.

Die unlängst veröffentlichte ESC-Leitlinie [3] stellt die Aktualisierung der vorherigen Fassung aus 2015 [4] dar. Die Überarbeitung geht dabei in verschiedener Hinsicht neue Wege. Neben der inhaltlichen Überarbeitung wurde der methodische Ansatz der neuen Leitlinie überarbeitet. Indem zahlreiche Algorithmen in übersichtlichen graphischen Darstellungen in den Leitlinientext aufgenommen wurden, steht die Überarbeitung für eine erhöhte Praxisnähe und zeigt die Entwicklung zu einem Handlungsleitfaden für die klinische Praxis auf. Einige Krankheitsentitäten bzw. Syndrome wie die frühe Repolarisation oder das Andersen-Tawil-Syndrom werden erstmals behandelt. Dieser Artikel gibt einen Überblick über wesentliche Neuerungen und wichtige Empfehlungen in der Akut- und Langzeitbehandlung ventrikulärer Arrhythmien.

## Akuttherapie ventrikulärer Arrhythmien

Unabhängig davon, ob sich ein Patient erstmalig mit ventrikulären Extrasystolen oder Tachykardien vorstellt, bildet die umfassende diagnostische Evaluation den Ausgangspunkt jeder Therapie. Die Standarddiagnostik umfasst neben einem Ruhe-EKG, sofern möglich, auch ein 12-Kanal-EKG der Arrhythmie sowie eine wenigstens orientierende Echokardiographie, insbesondere bei fehlendem Hinweis auf eine strukturelle Herzerkrankung. In der Akutsituation stehen Anamnese, körperliche Untersuchung sowie ein 12-Kanal-EKG im Vordergrund. Beachtenswert ist, dass reversible Ursachen wie eine Ischämie oder seltener Elektrolytstörungen für bis zu 50 % aller Fälle eines plötzlichen Herztods verantwortlich sind [5, 6]. Daraus folgt zwingend, dass sowohl die Untersuchung hinsichtlich möglicher reversibler Ursachen als auch deren unverzügliche Behandlung wichtiger Bestandteil der Akuttherapie ventrikulärer Arrhythmien sind.

Medikamenteninduzierte Arrhythmien treten insbesondere im Rahmen der Therapie mit QT_c_- und QRS verlängernden Pharmaka auf, spielen aber auch bei Medikamenten, die Elektrolytstörungen induzieren können, wie Schleifen- und Thiaziddiuretika eine Rolle. Bei Verdacht auf medikamenteninduzierte Arrhythmien sollte die Behandlung mit dem betreffenden Wirkstoff unverzüglich beendet werden.

Die Akutbehandlung ventrikulärer Arrhythmien richtet sich grundsätzlich nach der klinischen Präsentation und zugrundeliegender Ursache der Herzrhythmusstörung. Erwartungsgemäß besteht die Empfehlung zur unverzüglichen Einleitung von Reanimationsmaßnahmen inklusive Kardioversion bzw. Defibrillation bei hämodynamisch nicht tolerierten ventrikulären Arrhythmien fort. Neu ist hingegen die Klasse I-Empfehlung zur elektrischen Kardioversion auch im Fall einer hämodynamisch tolerierten ventrikulären Tachykardie (VT).

### Idiopathische ventrikuläre Extrasystolen und Tachykardien

**Figure 1:**
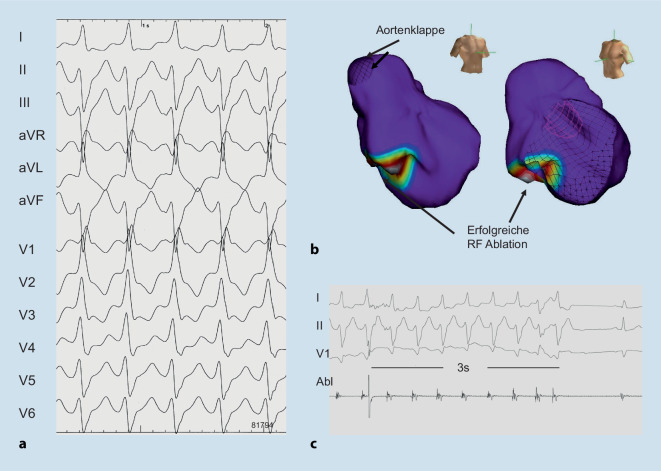

**Figure 2:**
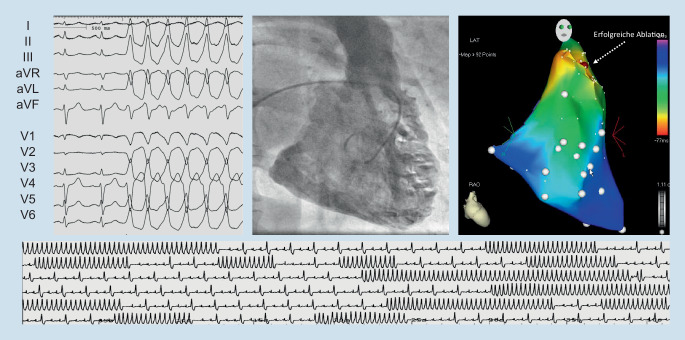

Unverändert empfiehlt die Leitlinie die Gabe von Verapamil im Fall einer bekannten linksfaszikulären VT, die im 12-Kanal-EKG typischerweise eine Rechtsschenkelblock-Morphologie mit superiorer Achse aufweist (Abb. 1). Bei Ausflusstrakttachykardie mit typischer Linksschenkelblock-Morphologie und inferiorer Achse (Abb. 2) werden weiterhin Betablocker empfohlen. Andere Antiarrhythmika, etwa Flecainid, Sotalol oder Ajmalin, können alternativ erwogen werden (Klasse IIb-Empfehlungen), wobei eine signifikante strukturelle Herzerkrankung nicht vorliegen sollte.

### Ventrikuläre Arrhythmien bei struktureller Herzerkrankung

Die Neufassung der Leitlinie empfiehlt zur antiarrhythmischen Akutbehandlung einer regelmäßigen Breitkomplextachykardie bei vermuteter oder bekannter struktureller Herzerkrankung als Neuerung primär Procainamid (Klasse IIa-Empfehlung). Dies resultiert aus den Ergebnissen der PROCAMIO-Studie, in der Procainamid im Vergleich zu Amiodaron mit weniger Nebenwirkungen bei höherer Rate einer frühen Terminierung der Arrhythmie assoziiert war [7]. Da Procainamid in Deutschland nicht zugelassen ist, hat diese Empfehlung für unsere Praxis jedoch kaum Bedeutung. Für den Einsatz des hingegen gut verfügbaren Ajmalins formuliert die aktuelle Leitlinie erstmals eine Klasse IIb-Empfehlung bei hämodynamisch tolerierter VT und Fehlen einer nicht näher bezeichneten „signifikanten strukturellen Herzerkrankung“. Da die Grunderkrankung bei Erstmanifestation einer monomorphen VT oft nicht bekannt ist, sollte Ajmalin deshalb erst nach echokardiographischem Ausschluss einer höhergradigen Einschränkung der linksventrikulären Ejektionsfraktion (LVEF) eingesetzt werden.

### Ventrikuläre Arrhythmien bei Ionenkanalerkrankungen

**Figure 3:**
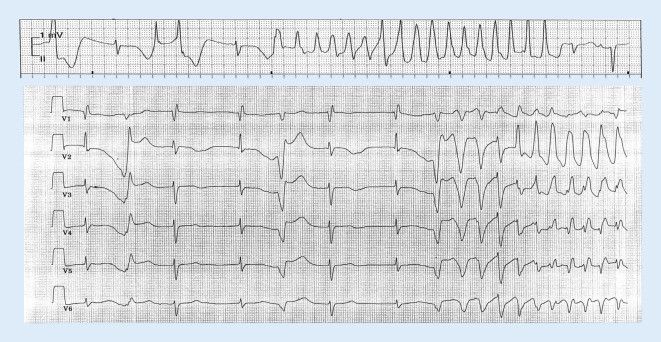

Beim langen QT-Syndrom können polymorphe Kammertachykardien vom Torsade-de-Pointes-Typ (TdP; Abb. 3) zu Synkopen und plötzlichem Herztod führen. Die i.v.-Gabe von Magnesium als potentem Kalziumkanalblocker unterdrückt selbst bei normaler Magnesiumkonzentration (0,8 und 1,1 mmol/l) TdP sehr effektiv [8], sodass die dahingehende Klasse I-Empfehlung, ebenso wie zur i.v.-Korrektur des Kaliumspiegels auf hochnormale Werte, fortbesteht. Dies gilt gleichermaßen für Patienten mit kongenitalem wie erworbenem Long-QT-Syndrom. Sind TdP bei einem erworbenen langen-QT-Syndrom trotz Magnesiumgabe und Korrektur anderer begünstigender Faktoren therapierefraktär, wird bei oftmals bradykardieassoziierten TdP und zur Verhinderung initiierender „Short-long-short“-Sequenzen eine passagere transvenöse Schrittmacherstimulation oder Isoproterenol empfohlen.

### Management des elektrischen Sturms

Die Neufassung der Leitlinie legt einen besonderen Schwerpunkt auf das Management des elektrischen Sturms, der formal durch ≥ 3 anhaltende Episoden ventrikulärer Arrhythmien binnen 24 h definiert ist. Er ist mit erheblicher psychischer Belastung der Betroffenen sowie einer erhöhten Mortalität assoziiert [9]. Therapeutisch wird ein schrittweiser Behandlungsansatz bestehend aus ICD-Kontrolle und ggf. -Reprogrammierung, medikamentöser antiarrhythmischer Therapie, Sedierung, Katheterablation und im Einzelfall ergänzender autonomer Modulation sowie mechanischer Kreislaufunterstützung empfohlen. Bei gehäuften ICD-Interventionen sollten initial inadäquate ICD-Therapien, die durch Oversensing, tachykard übergeleitetes Vorhofflimmern oder supraventrikuläre Tachykardien bedingt sein können, ausgeschlossen bzw. behandelt werden. Dies beinhaltet auch eine optimale Programmierung des ICD.

Bei Patienten mit struktureller Herzerkrankung steht die medikamentöse antiarrhythmische Therapie mit bevorzugt nichtselektiven Betablockern und Amiodaron unverändert im Vordergrund. Zur Minderung des Sympathikotonus und Reduktion der psychischen Belastung wird überdies eine Sedierung empfohlen. Für den Fall unaufhörlicher („incessant“) Tachykardien oder eines elektrischen Sturms aufgrund rezidivierender monomorpher VT wird eine rasche Katheterablation empfohlen (jeweils Klasse I-Empfehlungen). Für die Katheterablation ventrikulärer Extrasystolen (VES), die therapierefraktäre, rezidivierende Episoden polymorpher VT oder Kammerflimmern induzieren, besteht ebenfalls eine Indikation zur Ablation (Klasse IIa-Empfehlung).

Bleiben die medikamentöse antiarrhythmische Therapie und eine Katheterablation im elektrischen Sturm wirkungslos, kann neben tiefer Sedierung bzw. Intubationsnarkose eine Modulation des autonomen Nervensystems erwogen werden (Klasse IIb-Empfehlung). Im kardiogenen Schock kann zudem der Einsatz mechanischer Kreislaufunterstützungssysteme notwendig sein. Das in Deutschland nicht zugelassene Chinidin wird als Reservemittel bei Patienten mit koronarer Herzerkrankung (KHK) und rezidivierenden polymorphen, therapierefraktären Kammertachykardien erwähnt (beides Klasse IIb-Empfehlungen). Weitere relevante Änderungen in der Akuttherapie des elektrischen Sturms und bei wiederkehrenden ICD-Therapieabgaben betreffen insbesondere die primär elektrischen Erkrankungen. Erstmals wird der Einsatz von Chinidin bei idiopathischem Kammerflimmern sowohl in der Akuttherapie als auch in der Rezidivprophylaxe eines elektrischen Sturms empfohlen, akut kann auch Verapamil eingesetzt werden (beides Klasse IIa-Empfehlungen).

## Langzeitmanagement ventrikulärer Arrhythmien: von der Pharmako- zur Devicetherapie und Katheterablation

Ein prognostischer Nutzen einer antiarrhythmischen Pharmakotherapie ventrikulärer Arrhythmien ist weiterhin nicht bekannt. Gleichwohl haben Antiarrhythmika als ergänzende Therapie, insbesondere in der Behandlung symptomatischer rezidivierender ventrikulärer Arrhythmien, einen hohen Stellenwert. Die Leitlinie enthält eine umfassende Übersichtstabelle über die in der Akut- und Langzeittherapie ventrikulärer Arrhythmien häufig eingesetzten Antiarrhythmika. Die Bedeutung proarrhythmischer Nebenwirkungen von Antiarrhythmika, aber auch anderen Medikamenten, [10] greift die Leitlinie auf und führt erstmals Algorithmen zu Beginn und Fortführen einer Therapie mit Antiarrhythmika der Klasse I nach Vaughan Williamswie Flecainid und Propafenon sowie QT_c_-verlängernden Medikamenten ein. So wird für QT_c_-verlängernde Substanzen am Tag nach Therapiebeginn sowie 1–2 Wochen später und/oder nach jeder Dosissteigerung eine EKG-Kontrolle empfohlen. Ab einer QT_c_ > 0,5 s sollte eine besonders strenge Abwägung von Nutzen und Risiko und idealerweise eine Dosisreduktion oder Beendigung der Therapie erfolgen.

### Idiopathische ventrikuläre Extrasystolen und Tachykardien

In der Therapie idiopathischer VES und VT ist die interventionelle Therapie aufgewertet. So wird die Katheterablation symptomatischer VES und VT mit Ursprung im rechtsventrikulären Ausflusstrakt und linken Faszikel in der Neufassung der Leitlinie zur Erstlinientherapie (Klasse I-Empfehlung). Medikamentöse Therapieoptionen, d. h. Betablocker, Kalziumkanalantagonisten oder Flecainid, werden zur Zweitlinientherapie (Tab. 1). Da die Datenlage für eine Katheterablation anderer idiopathischer VES und Tachykardien weniger eindeutig [10] und das periprozedurale Komplikationsrisiko höher ist, ist die Empfehlung zur Katheterablation in dieser Situation schwächer (Klasse IIa-Empfehlung), während eine Behandlung mit Betablockern oder Kalziumkanalantagonisten Erstlinientherapie ist (Klasse I-Empfehlung). Der Einsatz von Amiodaron in der Erstlinientherapie idiopathischer VES und VT wird bei normaler LVEF ausdrücklich nicht empfohlen (Klasse III-Empfehlung).

**Table 1:** 

	Ablation	Betablocker	Kalziumkanalantagonisten	Antiarrhythmika
Symptomatische RVOT/faszikuläre VES/VT, normale LV-Funktion	Klasse I	Klasse IIa	Klasse IIa	Flecainid (Klasse IIa)
				Amiodaron (Klasse III)
RVOT/faszikuläre VES/VT + LV-Dysfunktion	Klasse I	Klasse IIa	Klasse III	Flecainid (Klasse IIa^a^)
				Amiodaron (Klasse IIa)
Symptomatische VES/VT anderen Ursprungs, normale LV-Funktion	Klasse IIa	Klasse I	Klasse I	Flecainid (Klasse IIa)
				Amiodaron (Klasse III)
VES/VT anderen Ursprungs + LV-Dysfunktion	Klasse I	Klasse IIa	Klasse III	Flecainid (Klasse IIa^a^)
				Amiodaron (Klasse IIa)

*LV-Funktion* linksventrikuläre Funktion, *RVOT* rechtsventrikulärer Ausflusstrakt, *VES* ventrikuläre Extrasystolen, *VT* ventrikuläre Tachykardie

^a^nur bei ausgewählten Patienten mit moderater LV-Dysfunktion

Erstmals wird auch eine Ablation asymptomatischer idiopathischer VES ab einer VES-Last ≥ 20 % empfohlen (Klasse-IIb-Empfehlung). Eine gehäufte ventrikuläre Extrasystolie kann reversible Ursache einer linksventrikulären Dysfunktion bei Patienten mit ansonsten strukturell unauffälligem Herzen sein, wofür das Risiko wahrscheinlich ab einer VES-Last > 20 % erhöht ist [11]. Besteht der Verdacht auf eine VES-induzierte Einschränkung der linksventrikulären Funktion, wird ebenfalls eine Katheterablation empfohlen. Auch hier spricht sich die Leitlinie für den Einsatz von Antiarrhythmika lediglich als Zweitlinientherapie aus (Klasse IIa-Empfehlung).

### Ventrikuläre Arrhythmien bei struktureller Herzerkrankung

#### Pharmakotherapie.

Grundlage des Langzeitmanagements von Patienten mit reduzierter linksventrikulärer Pumpfunktion und ventrikulären Arrhythmien auf dem Boden einer strukturellen Herzerkrankung ist eine medikamentöse Herzinsuffizienztherapie. Die Leitlinie ergänzt die Empfehlungen diesbezüglich entsprechend der aktuellen Leitlinien zur Herzinsuffizienztherapie [12] um SGLT2-Inhibitoren zusätzlich zu Betablockern, ACE-Inhibitoren bzw. Angiotensinrezeptorblockern/Neprilysin-Inhibitoren und Mineralkortikoidantagonisten.

#### Devicetherapie.

Unverändert ist die ICD-Therapie integraler Bestandteil der Sekundärprävention des plötzlichen Herztods bei Patienten mit dokumentiertem Kammerflimmern oder hämodynamisch nichttolerierter VT [13]. Ebenso hat die Devicetherapie in der Primärprävention des plötzlichen Herztods bei Patienten mit deutlich erhöhtem Risiko ventrikulärer Arrhythmien, trotz erheblicher Verbesserungen der medikamentösen Herzinsuffizienztherapie, weiterhin große Bedeutung. Als Neuerung enthält die Leitlinie mit Verweis auf aktuelle Konsenspapiere zu diesem Thema [14, 15] erstmals konkrete Empfehlungen zur optimalen Deviceprogrammierung, die auch die vergleichsweise jungen subkutanen ICD-Systeme einschließen.

Kriterien zur primärprophylaktischen ICD-Implantation werden in den aktuellen Leitlinien ausführlich diskutiert. Insgesamt wird an der LVEF als bedeutendem Risikomarker festgehalten. So besteht bei KHK-Patienten mit LVEF ≤ 35 % trotz mindestens 3 Monaten optimaler medikamentöser Therapie und Herzinsuffizienzsymptomatik (NYHA II–III) eine Klasse-I-Empfehlung zur primärprophylaktischen ICD-Implantation. Die wenigen weiteren Klasse-I-Indikationen hierfür sind in Tab. 2 aufgeführt. Erstmals spricht die Leitlinie überdies eine Klasse IIa-Empfehlung zur ICD-Implantation bei KHK-Patienten mit einer LVEF ≤ 30 % ohne Herzinsuffizienz aus. Bei dilatativer Kardiomyopathie (DCM), LVEF ≤ 35 % trotz mindestens 3 Monaten optimaler medikamentöser Therapie und Herzinsuffizienzsymptomatik (NYHA II–III) wird die Empfehlung zur primärprophylaktischen ICD-Implantation hingegen, basierend auf den Ergebnissen der DANISH-Studie [16], von einer Klasse I- auf eine IIa-Empfehlung abgewertet. Gleichwohl wird auch bei DCM-Patienten mit einer nur leicht- oder mittelgradig reduzierten LVEF < 50 %, die mehr als einen weiteren Risikofaktor wie Synkope, signifikante Kontrastmittelanreicherungen in der kardialen MRT, bestimmte pathogene Mutationen oder Induzierbarkeit anhaltender VT aufweisen, eine ICD-Implantation empfohlen (Klasse IIa-Empfehlung). Diese Entwicklung deutet auf eine Abkehr von der LVEF als alleinigem Risikomarker hin und spiegelt den Bedeutungsgewinn *neuer* diagnostischer Methoden wie genetischer Testung und der kardialen MRT wider. Die programmierte elektrische Stimulation zur Risikostratifikation erlebt mit der Neufassung an verschiedenen Stellen eine Aufwertung. So wird eine ICD-Implantation bei KHK mit LVEF ≤ 40 % und dokumentierter nichtanhaltender VT empfohlen, falls eine anhaltende monomorphe VT induzierbar ist (Klasse IIa-Empfehlung).

**Table 2:** 

*Strukturelle Herzerkrankung*	*Empfehlungsgrad*
Ischämische Kardiomyopathie + LVEF ≤ 35 % trotz 3 Monaten OMT + NYHA II-III	I
Kardiale Sarkoidose + LVEF ≤ 35 %	I
Angeborene Herzerkrankung mit biventrikulärer Physiologie und systemischem linken Ventrikel + LVEF ≤ 35 % trotz 3 Monaten OMT + NYHA II–III	I
*Primär elektrische Erkrankungen*	*Empfehlungsgrad*
Langes QT-Syndrom + Symptomatik (u. a. rhythmogene Synkope^a^) während Betablocker- und ggf. genotypspezifischer Therapie	I

LVEF linksventrikuläre Ejektionsfraktion, OMT optimale medikamentöse Therapie, NYHA New York Heart Association

^a^Da die Symptomatik formal auch auf anhaltenden Rhythmusstörungen (z. B. TdP > 30 s; hämodynamisch nichttolerierte VT) beruhen kann, könnte diese Empfehlung auch der Sekundärprophylaxe zugeordnet werden

#### Katheterablation.

Die Katheterablation gewinnt mit der neuen Leitlinie auch im Kontext struktureller Herzerkrankungen an Bedeutung. Aktuelle Ablationszahlen in Deutschland spiegeln dies wider [17]. So wird für KHK-Patienten mit erhaltener LVEF ≥ 40 % und hämodynamisch tolerierter VT erstmals eine Klasse-IIa-Empfehlung zur Katheterablation als Alternative zur ICD-Implantation ausgesprochen, wenngleich prospektive Daten bisher fehlen. Bemerkenswert ist, dass 3 Studien zur sekundärprophylaktischen ICD-Therapie für dieses Patientenkollektiv keinen Überlebensvorteil zeigen konnten [13]. Eine weitere bedeutsame Neuerung stellt die Klasse I-Empfehlung zur Katheterablation bei rezidivierenden VT unter chronischer Amiodarontherapie bei KHK-Patienten dar, die aus den Ergebnissen der VANISH-Studie [18] resultiert. Für den Fall rezidivierender symptomatischer VT oder ICD-Schocks trotz Betablocker- oder Sotaloltherapie sollte ebenfalls eine Katheterablation erwogen werden (Klasse IIa-Empfehlung). Neuere Studien wie die PARTITA-Studie [19], die auf einen prognostischen Nutzen einer früheren Katheterablation bei ICD-Trägern hindeuten, konnten in der Leitlinie aufgrund der Veröffentlichung nach Fertigstellen der Leitlinie nicht berücksichtigt werden, sodass der *ideale* Zeitpunkt einer Ablationstherapie bei Patienten mit struktureller Herzerkrankung Gegenstand intensiver Diskussion ist [20]. Wenngleich die Datenlage für die Katheterablation bei Patienten mit DCM nicht so positiv ist [21, 22], ist diese für Kontraindikation oder Unverträglichkeit von Antiarrhythmika oder Ineffektivität aufgewertet worden (Klasse IIa-Empfehlung).

### Ventrikuläre Arrhythmien bei Ionenkanalerkrankungen

Analog zu den *klassischen* strukturellen Herzerkrankungen wird eine sekundärprophylaktische ICD-Implantation bei primär elektrischen Erkrankungen nach überlebtem Herzstillstand bzw. bei Patienten mit Brugada- oder einem kurzen QT-Syndrom nach Dokumentation einer anhaltenden VT empfohlen. Die Risikostratifikation bei Patienten mit Ionenkanalerkrankungen bleibt eine Herausforderung [23]. Ähnlich zu Risikokalkulatoren für hypertrophe Kardiomyopathie [24] und Laminopathien [25] wurde in die neue Leitlinie ein Risikokalkulator für das lange QT-Syndrom (LQTS) aufgenommen [26]. Für Patienten mit LQTS, bei denen unter Betablocker- und evtl. genotypspezifischer Therapie Synkopen oder hämodynamisch nicht tolerierte VT auftreten, wurde die Empfehlung zur ICD-Implantation verstärkt (nun Klasse I). Analog enthält die Leitlinie neue Empfehlungen zur primärprophylaktischen ICD-Therapie nach rhythmogener Synkope bei kurzem QT-Syndrom (Klasse IIa-Empfehlung).

In der antiarrhythmischen Pharmakotherapie von Patienten mit Ionenkanalerkrankungen fallen zahlreiche neue Empfehlungen ins Auge: Die Neufassung der Leitlinie empfiehlt eine Betablockertherapie, idealerweise mit den nichtselektiven Betablockern Nadolol (in Deutschland leider nicht zugelassen) und Propranolol, bei Patienten mit LQTS und QT_c_-Verlängerung, aber auch bei normaler QT_c_-Zeit, sofern ein LQTS genetisch nachgewiesen wurde. Überdies wird Mexiletin als genotypspezifische Therapie bei LQT3 erstmals empfohlen. Betablocker und/oder Flecainid werden auch für Patienten mit Andersen-Tawil-Syndrom (LQT7) empfohlen (Klasse IIa). Die Klasse I-Empfehlung zur Betablockertherapie bei allen Patienten mit klinisch diagnostizierter katecholaminerger polymorpher ventrikulärer Tachykardie (CPVT) besteht fort, allerdings bevorzugt die Neufassung der Leitlinie auch hier den Einsatz nichtselektiver Betablocker; bei asymptomatischen Patienten mit genetischem Nachweis CPVT-typischer Mutationen ist die Empfehlung schwächer (Klasse IIa). Bei rezidivierenden Synkopen, polymorphen bzw. bidirektionalen VT oder belastungsinduzierten VES unter Betablockertherapie sollte Flecainid ergänzend eingesetzt werden ([27]; Klasse IIa-Empfehlung). Chinidin (s. oben) kann bei Patienten mit Brugada-Syndrom, einem „early repolarization syndrome“ und asymptomatischen Patienten mit positiver Familienanamnese für einen plötzlichen Herztod bei kurzem QT-Syndrom im Einzelfall als Alternative zur ICD-Implantation erwogen werden (Klasse IIb-Empfehlungen).

Die Katheterablation ist bei Ionenkanalerkrankungen v. a. bei rezidivierenden, therapierefraktären Arrhythmien von Bedeutung. Bereits die vorherige Leitlinie beinhaltete eine Klasse-IIa-Empfehlung für die Ablation häufiger VES bei Patienten mit idiopathischem Kammerflimmern und Brugada-Syndrom, wenn diese rezidivierende Episoden von Kammerflimmern auslösen. Die Neufassung formuliert eine solche Empfehlung erstmals auch für Patienten mit „early repolarization syndrome“.

## Fazit

Gemessen an den Empfehlungen aus dem Jahr 2015 [4, 28] enthält die Neufassung der ESC-Leitlinie zahlreiche neue Empfehlungen und Änderungen für das Akut- und Langzeitmanagement von Patienten mit ventrikulären Arrhythmien. Sieben Jahre nach der Veröffentlichung ist auf Grundlage der aktuellen Evidenz eine praxisnahe Aktualisierung entstanden, die als Nachschlagewerk für den Kliniker alle wichtigen Informationen zusammenfasst. Jenseits inhaltlicher Neuerungen machen zahlreiche Übersichtstabellen, Flussdiagramme und neue Abschnitte die neue Leitlinie zu einem auf die klinische Praxis zugeschnittenen, anwenderfreundlichen Nachschlagewerk, das auch besondere klinische Situationen wie Rhythmusstörungen in der Schwangerschaft [29] oder im Zusammenhang mit Sport umfasst.

Eine Neuerung in der Akutbehandlung stellt die Empfehlung zur Elektrokardioversion als Erstlinientherapie bei hämodynamisch tolerierter VT dar. Während die Empfehlungen zur pharmakologischen Therapie idiopathischer VT im Wesentlichen unangetastet bleiben, wird das in Deutschland nicht verfügbare Procainamid zum Antiarrhythmikum der ersten Wahl in der Akuttherapie monomorpher VT bei struktureller Herzerkrankung. In erfahrener Hand stellt Ajmalin in Deutschland eine Alternative dar [30]. Amiodaron kann alternativ eingesetzt werden, wenngleich die Leitlinie hierfür nur noch eine zurückhaltende Empfehlung formuliert. Insgesamt legt die Überarbeitung einen besonderen Fokus auf das Management des elektrischen Sturms. Bei medikamentenrefraktärem elektrischem Sturm fordert die Leitlinie einen schrittweisen Behandlungsansatz, der neben Sedierung und Amiodaron die Katheterablation, Kreislaufunterstützungssysteme und Optionen zur Modulation des autonomen Nervensystems umfasst.

Im Langzeitmanagement ventrikulärer Arrhythmien werden die Empfehlungen zur optimalen medikamentösen Behandlung der Grunderkrankung an die Empfehlungen der Herzinsuffizienzleitlinie angeglichen. Die Neufassung empfiehlt den Einsatz der nichtselektiven Betablocker Propranolol und Nadolol nicht nur bei langem QT-Syndrom, sondern auch bei der CPVT. Erstmals wird mit Mexiletin bei LQT3 auch eine genotypspezifische Therapie empfohlen. Bemerkenswert ist insgesamt die deutliche Aufwertung der Katheterablation. Diese wird bei Patienten mit idiopathischen VES und VT teilweise zur Erstlinientherapie. Die Katheterablation erhält zudem Vorrang gegenüber einer Eskalation der medikamentösen antiarrhythmischen Therapie bei KHK-Patienten mit rezidivierenden Kammertachykardien.

## Fazit für die Praxis

- Die Neufassung der ESC-Leitlinie enthält zahlreiche neue Empfehlungen und Änderungen für das Akut- und Langzeitmanagement ventrikulärer Arrhythmien
- Im Akutmanagement anhaltender monomorpher VT gewinnt die Elektrokardioversion an Bedeutung, aber Ajmalin als Alternative zum Amiodaron hat erstmals Eingang in die Empfehlungen gefunden
- Im elektrischen Sturm ist ein schrittweiser Behandlungsansatz der neben Sedierung und Amiodaron die Katheterablation, Kreislaufunterstützungssysteme und Optionen zur Modulation des autonomen Nervensystems umfasst, gefragt
- Empfehlungen zur medikamentösen Langzeittherapie sind an die Herzinsuffizienz-Leitlinien angeglichen
- Die Katheterablation gewinnt insb. in der Therapie idiopathischer VES, aber auch bei rezidivierenden Arrhythmien unter Amiodarontherapie bei Patienten mit KHK an Bedeutung
- Zahlreiche Übersichtstabellen, Flussdiagramme sowie neue und überarbeitete Inhalte machen die Leitlinie zu einem praxisnahen, anwenderfreundlichen Nachschlagewerk
